# Efficient generation of region-specific forebrain neurons from human pluripotent stem cells under highly defined condition

**DOI:** 10.1038/srep18550

**Published:** 2015-12-16

**Authors:** Fang Yuan, Kai-Heng Fang, Shi-Ying Cao, Zhuang-Yin Qu, Qi Li, Robert Krencik, Min Xu, Anita Bhattacharyya, Yu-Wen Su, Dong-Ya Zhu, Yan Liu

**Affiliations:** 1 Institute for Stem Cell and Neural Regeneration, School of Pharmacy, Nanjing Medical University, Nanjing, China; 2 Department of Ophthalmology, University of California, San Francisco, San Francisco, CA 94143, USA; 3 Waisman Center, University of Wisconsin, Madison, WI, USA

## Abstract

Human pluripotent stem cells (hPSCs) have potential to differentiate to unlimited number of neural cells, which provide powerful tools for neural regeneration. To date, most reported protocols were established with an animal feeder system. However, cells derived on this system are inappropriate for the translation to clinical applications because of the introduction of xenogenetic factors. In this study, we provided an optimized paradigm to generate region-specific forebrain neurons from hPSCs under a defined system. We assessed five conditions and found that a vitronectin-coated substrate was the most efficient method to differentiate hPSCs to neurons and astrocytes. More importantly, by applying different doses of purmorphamine, a small-molecule agonist of sonic hedgehog signaling, hPSCs were differentiated to different region-specific forebrain neuron subtypes, including glutamatergic neurons, striatal medium spiny neurons, and GABA interneurons. Our study offers a highly defined system without exogenetic factors to produce human neurons and astrocytes for translational medical studies, including cell therapy and stem cell-based drug discovery.

The forebrain is an essential functional region in the central nervous system. Cortical excitatory glutamatergic neurons, inhibitory striatal medium spiny neurons (MSNs) and GABA interneurons of the cortex are three major neuronal subtypes in forebrain. Dysfunction of forebrain neurons cause severe neurological diseases, such as Alzheimer’s disease^1^, Huntington’s disease^2^ and Schizophrenia^3^. Availability of human forebrain neurons *in vitro* offers an opportunity for cellular-based therapy and drug discoveries for these diseases. Human pluripotent stem cells (hPSCs) hold the potential to generate unlimited number of functional neurons. Breakthroughs have been made on direct neural differentiation from hPSCs over the last decade^4^,^5^,^6^,^7^,^8^,^9^. Ours and other’s studies showed that cortical neurons^9^, MSNs^10^ and cortical GABA interneurons^11^,^12^,^13^ were effectively differentiated from hPSCs, respectively. However, most reported protocols relied on maintenance of hPSCs with undefined components including irradiated mouse embryonic fibroblasts (MEF) or animal ingredients. Feeder cells and other undefined components introduced exogenous sources into human-derived system, which may hamper stem cell applications for clinical cell therapy and human cell-based pharmaceutical studies.

Recently, chemically defined feeder-free systems for hPSC culture have been reported, which increases the safety of hPSCs and derivatives for future application^14^,^15^. The use of xeno-free medium for hPSC culture is in complete absence of animal ingredients, offering a xeno-free system for the studies of self-renewal and pluripotency maintenance, however, limited studies were performed on the direct lineage differentiation of hPSCs under xeno-free conditions^16^,^17^. Moreover, efficient differentiation of hPSCs to specific forebrain neuronal lineages under xeno-free system has not been well-established. Thus, aiming at promoting clinical application of cell therapy, an optimized system for forebrain neural differentiation excluding animal component contamination is desired.

In this study, we provide an optimized approach for differentiation of hPSCs to region-specific forebrain neurons under xeno-free system. In comparison with five conditions, we found that hPSCs on a vitronectin-coated surface and dissociated with dispase was the most efficient condition to differentiate to neurons. By using the optimized conditions and embryoid body (EB) protocol, hPSCs were robustly differentiated to forebrain neurons (FOXG1 + ) with high efficiency (>90%). Furthermore, we specified hPSCs to region-specific forebrain neurons by applying different doses of purmorphamine (Pur), a substitute for SHH. The hPSC-derived neuroepithelial cells were patterned to nearly pure dorsal forebrain (PAX6 + ), lateral ganglionic eminence (MEIS2 + ), or medial ganglionic eminence (NKX2.1 + ) progenitors under xeno-free condition, which further matured to cerebral cortex glutamatergic neurons, MSNs, or GABA interneurons, respectively. Our current research provides an opportunity that effectively generates region-specific neurons under xeno-free condition,moving forward hPSC-derived neurons for translational applications concerning the treatment of forebrain neuron-associated diseases.

## Results

### Vitronectin improves the efficiency of neural differentiation from hPSC under defined conditions

In order to optimize culture conditions, hPSCs (hESC, H9; iPSC, DS-AG1, DS-AG2U^18^) were maintained on matrigel or a vitronectin-coated substrate in E8 medium^14^ for more than 20 passages. hPSCs were passaged with EDTA, and re-seeded at the density of 1×10^5^ in one well of 6 well plate We observed that hPSCs maintained undifferentiated morphology with round and clear edges in control conditions. In contrast, hPSCs showed irregular morphology with the treatment of the commonly used ROCK pathway inhibitor Y27632, and migrating cells were observed at the edge of colonies (Supplementary Fig. S1a, S1b). However, all the cells in the colony uniform expressed NANOG and SOX2 with or without the treatment of Y27632 (Supplementary Fig. S1c), and all colonies were positive in alkaline phosphatase staining as a test for pluripotency (Supplementary Fig. S1d). The pluripotent characteristics of hPSCs after 19 passages on feeder free culture were confirmed by SOX2 and NANOG immunostaining (Supplementary Fig. S1e). Together, this data showed that E8 medium maintains pluripotency of hPSCs for a long term.

**Figure 1:**
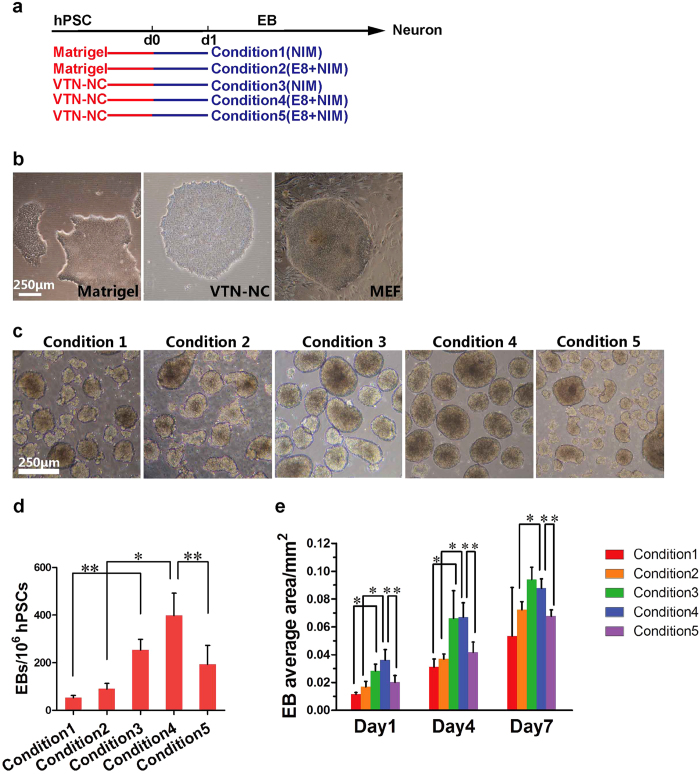
hPSCs cultured on vitronectin-coated substrate yielded highly efficient EB formation. (**a**) Schematic of differentiating hPSCs to EBs in different conditions. (**b**) hPSCs cultured on matrigel, vitronectin (VTN-NC) and MEF (mouse embryonic fibroblasts). Scale bar, 250 μm. (**c**) EB formation under different conditions starting on d1. Scale bar, 250 μm. (**d**) Quantifications of EB numbers starting from 1×10^6^ hPSCs under different condition systems. *p < 0.05. Data are presented as mean ± s.e.m. (**e**) Quantifications of EB average area under different condition systems. *p < 0.05. Data are presented as mean ± s.e.m.

To explore the best conditions for neural differentiation, we compared five paradigms which were described in Table 1 (Fig. 1a). The major two types of substrates were matrigel (condition 1 and 2) and vitronectin (condition 3, 4 and 5). Matrigel is a prevalent substrate for feeder free culture protocols^19^,^20^,^21^, while vitronectin was introduced by xeno-free culture with essential 8 medium^14^. We observed the morphology of hPSCs colonies on matrigel was irregular. In constrast, hPSCs on vitronectin were round and similar to the morphology that on MEF (Fig. 1b,Supplementary Fig. S1a, b).

Next, we differentiated hPSCs to neural epithelial cells under the five conditions (Fig. 1a, Supplementary Fig. S2a), following embryoid body (EB) formation protocols^4^. At the floating EB stage, EBs were homogeneous and round under condition 3 and 4, but showed fragmentary structure under other conditions (H9 in Fig. 1c, DS1 and DS2U in Supplementary Fig. S2b,c). Beginning with 1×10^6^ hPSCs, the number of EBs was more than 300 under condition 4 (vitronectin), but was less than 50 under condition 1 (matrigel) (Fig. 1d). Furthermore, the EBs in condition 3 and 4 (on vitronectin) showed larger size in comparison to condition 1 and 2 (on matrigel) (Fig. 1e). The results indicated that hPSCs cultured on vitronectin showed a higher efficiency to form EBs than the hPSCs cultured on matrigel.

**Figure 2:**
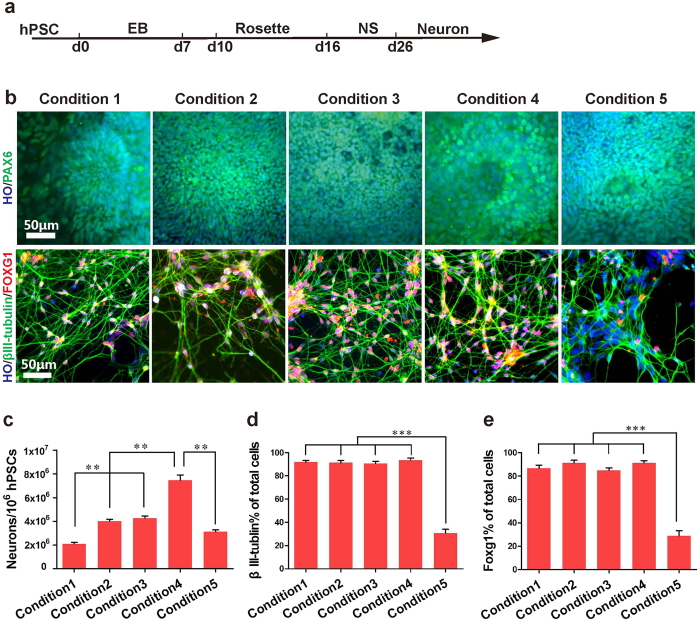
Optimized xeno-free condition yields highly efficient production of neurons. (**a**) Timeline of differentiation of hPSCs to neurons. (**b**) Upper panel: Pax6 + neuralepithial cells were observed from d10, with no significant differences in condition 1, 2, 3 and 4. Pax6 positive cells in condition 5 were significant decreased compared to other conditions. Lower panel: βIII-tublin + and FOXG1 + were expressed from d22, with similar levels in condition 1, 2, 3, and 4; while βIII-tublin + positive cells in condition 5 were significant decreased. Scale bar, 50 μm. (**c**) Quantifications of neuron numbers from each condition at d30 beginning with 1×10^6^ hPSCs. *p < 0.05. Data are presented as mean ± s.e.m. (**d**) Quantifications of βIII-tubulin + cells from different conditions. *p < 0.05; **p < 0.01; ***p < 0.001. Data are presented as mean ± s.e.m. (**e**) Quantifications of FOXG1 + cells from different conditions. *p < 0.05; **p < 0.01; ***p < 0.001. Data are presented as mean ± s.e.m.

At day 10 of differentiation (d10), more than 90% of cells expressed the neuroepithelial marker PAX6 under condition 1–4 (Fig. 2a,b). However, we observed less rosette formation in condition 5 (hPSCs dissociated by EDTA). At d26, more than 90% of differentiated cells were βIII-tubulin + and FOXG1 + under conditions 1, 2, 3 and 4,demonstrating a forebrain neuronal fate (Fig. 2b–d). In contrast, only 30% of differentiated cells were neurons under condition 5. The results suggest that hPSCs detached by dispase were more efficient for neural differentiation than EDTA. Although hPSCs under conditions 1–4 generated neurons with a similar neural differentiation percentage, the yield of neuron numbers was different. More than 7.5×10^6^ ± 0.5×10^6^ neurons were generated from 10^6^ hPSCs in condition 4, while only 2.1×10^6^ ± 0.1×10^6^ neurons were produced from the same number of hPSCs in condition 1 (Fig. 2c). Therefore, hPSCs cultured in condition 4 (vitronectin coated surface + dispase dissociated) was the most competent for neural differentiation. Vitronectin is a xeno-free substrate, whereas matrigel contains mouse ingredients^20^,^21^. Hence, we applied vitronectin as the substrate for all the attachment procedures, including attachments of rosettes and neurons. The components utilized in our defined system were listed in Supplementary Table S2.

### Cortical neurons are generated efficiently under xeno-free conditions

Previous neural differentiation protocols established on feeder cells showed that hPSCs could be patterned to a diverse number of neuronal subtypes by the treatment of specific morphogens^12^,^22^,^23^,^24^. Thus, an effective differentiation system is necessary to adapt xeno-free conditions to pattern neurons with region-specific information. To investigate whether the current xeno-free neural differentiation system might produce multiple forebrain neurons, we treated neuroepithelial cells at d10 with different concentrations of Pur, a small-molecule agonist of SHH signaling that inducing neurons with ventral fate^11^,^25^. Since condition 4 was the most efficient neural differentiation combination under xeno-free system, we thus far used as the optimized protocol for the following experiments.

**Figure 3:**
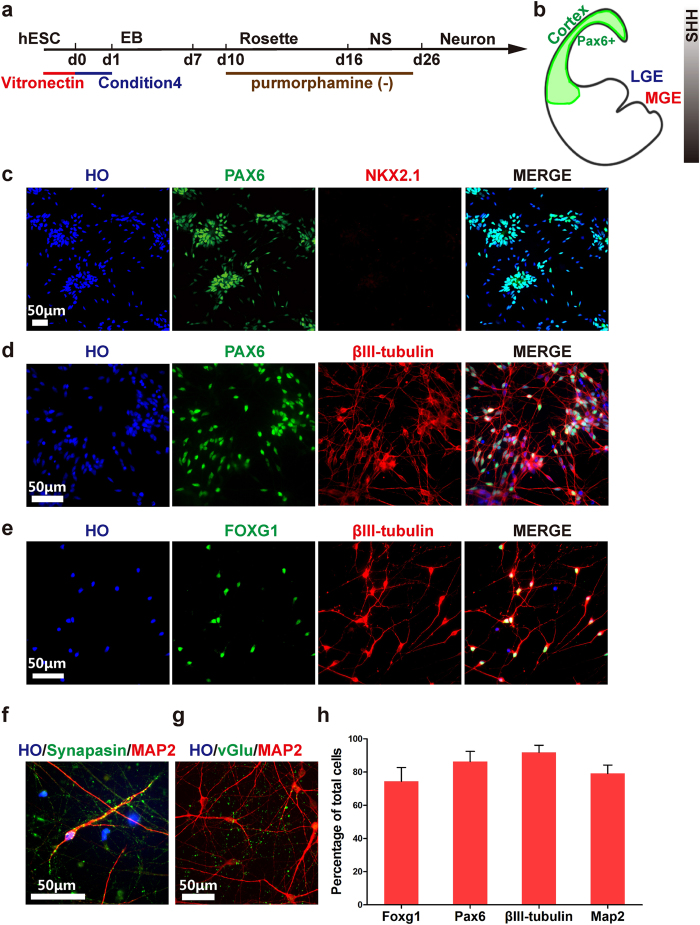
Directed differentiation of hPSCs to cortical neurons in a xeno-free system. (**a**) Timeline of direct differentiation of hPSCs to cortical neurons in a xeno-free system by using optimized condition 4. No purmorphamine treated in this condition. (**b**) A schematic of a coronal section of a human embryo with cerebral cortex (green) expressing marker PAX6. (**c**) At d22, nearly all cortical progenitors expressed dorsal forebrain marker PAX6 but did not express MGE marker NKX2.1. Scale bar, 50 μm. (**d**) At d25, 86.3 ± 1.6% of cells co-expressed PAX6 and βIII-tubulin, showing dorsal identity. Scale bar, 50 μm. (**e**) At d25, 74.4 ± 3.7% of total cells expressed βIII-tubulin + and FOXG1, showing forebrain identity. Scale bar, 50 μm. (**f**) At d45, neurons became mature and expressed mature neuronal marker MAP2 and pre-synaptic marker synapsin. Scale bar, 50 μm. (**g**) At d65, neurons expressed neuronal excitatory transmitter marker vGlut (vGlu), showing glutamatergic neuron identity. Scale bar, 50 μm. (**h**) Quantifications of FOXG1, Pax6, βIII-tubulin and MAP2 positive cells of total cells.

We first investigated whether hPSCs have the capability to differentiate to cortical neurons under xeno-free conditions. Glutamatergic neurons are the major cortical neuronal type and originate from dorsal telencephalic area that receives limited SHH signals^24^ (Fig. 3b, Supplementary Fig. S3a). Therefore, we did not treat the d10 rosettes with Pur as previously described^9^ (Fig. 3a). At d25, 86.3 ± 1.5% of cells expressed dorsal telencephalic marker PAX6, but the medial ganglionic eminence (MGE) marker NKX2.1 was not detected, indicating a cortical progenitor fate (Fig. 3c). Also, 77.5 ± 3.7% of cells expressed forebrain fate marker FOXG1 (Fig. 3e), and 86.3 ± 1.6% of cells expressed neuronal marker βIII-tubulin at d28 together with PAX6, demonstrating that the differentiated neurons have a cortical characteristic phenotype (Fig. 3d). The cortical neuronal differentiation from two iPSC lines showed a similar efficiency in the absence of xenogenetic materials (Supplementary Fig. S 3b–e). Neurons gradually became synapsin and MAP2 double positive (79 ± 1.8%) at d45, displaying indicators of maturation and formation of neuronal connections (Fig. 3f). After d60, neurons started to express a mature glutamatergic neuron marker vGLUT (Fig. 3g), indicating the glutamatergic neuronal fate. Taken together, mature cortical neurons, including glutamatergic neurons, were produced by a default morphogen-free differentiation in a defined system.

### Striatal medium spiny GABAergic neurons were efficiently generated under xeno-free condition

**Figure 4:**
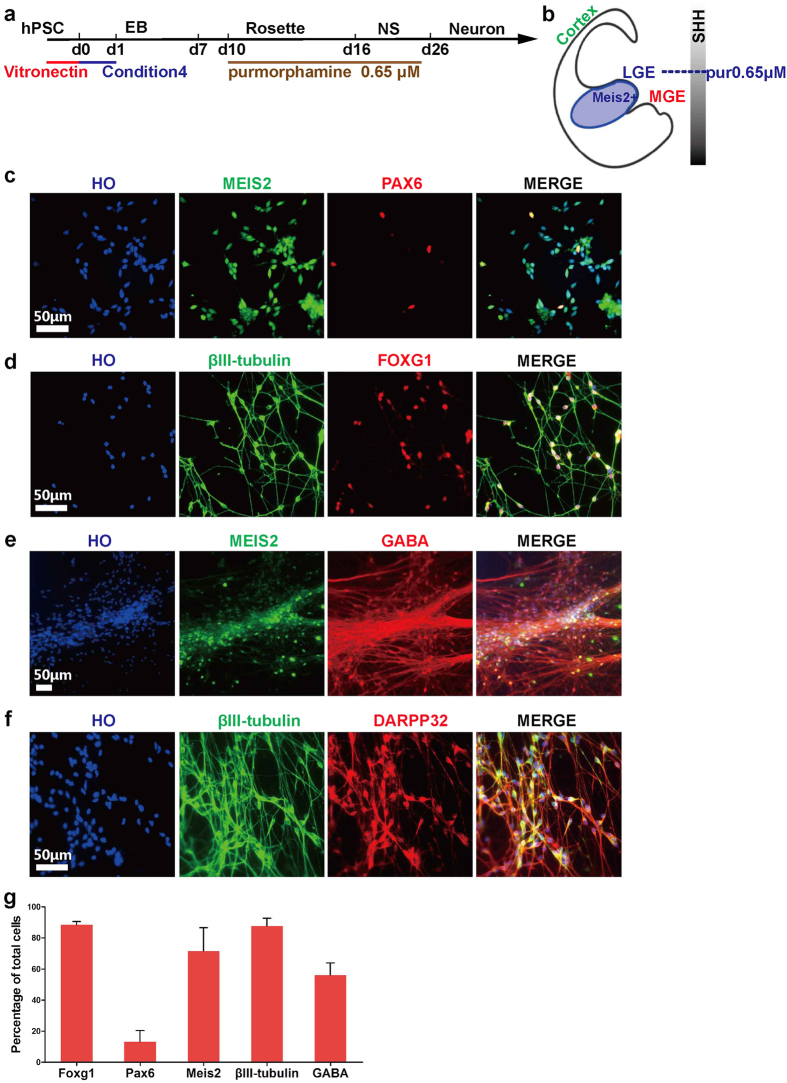
Directed differentiation of hPSCs to striatal medium spiny neurons in a xeno-free system. (**a**) Timeline of direct differentiation of hPSCs to striatal medium spiny neurons in a xeno-free system by using optimized condition 4 with low dosage of purmorphamine. (**b**) A schematic showing coronal section of a human embryo with LGE patterning (blue) containing cells expressing marker MEIS2. (**c**) At d22, 71.7 ± 3.7% of cells expressed MESI2, but only 13.2 ± 3.0% of cells expressed PAX6, demonstrating cells with LGE fate. Scale bar, 50 μm. (**d**) At d25, nearly all cells expressed FOXG1 and βIII-tubulin. Scale bar, 50 μm. (**e**) At d35, GABA + neurons co-expressed MEIS2. Scale bar, 50 μm. (**f**) At d35, most neurons expressed striatal projection neuron marker DARPP32. Scale bar, 50 μm. (**g**) Quantification of FOXG1 + , PAX6 + , MEIS2 + , βIII-tubulin + and GABA + cells of total cells.

MSNs are associated with many neurological degenerative disorders, such as Huntington’s disease. MSNs are the major projection neurons in striatum and originate from LGE^26^, which receives low concentrations of SHH during neural development (Fig. 4a). To generate MSNs from hPSCs in the absence of xenogenetic materials, we treated d10 rosettes with 0.65 μM of Pur as previously described^10^ (Fig. 4b). At d25, 71.7 ± 3.7% of cells expressed a marker for LGE progenitors, MEIS2. In comparison with cortical progenitors, moderate treatment of Pur dramatically decreased the expression of PAX6 (Fig. 4c). 10 days later, 87.7 ± 2.3% of cells were βIII-tubulin/FOXG1 double positive, demonstrating a forebrain neuron identity (Fig. 4d). After d45, 56.2 ± 3.5% of cells co-expressed GABA and MEIS2, demonstrating that GABA neurons were of LGE identity (Fig. 4e). The majority of those cells were DARPP32 positive, showing a MSN projection neuron fate (Fig. 4f). Thus, striatal MSNs were able to be efficiently generated under xeno-free conditions.

### High purity of cortical GABAergic interneurons were produced from hPSCs under xeno-free condition

**Figure 5:**
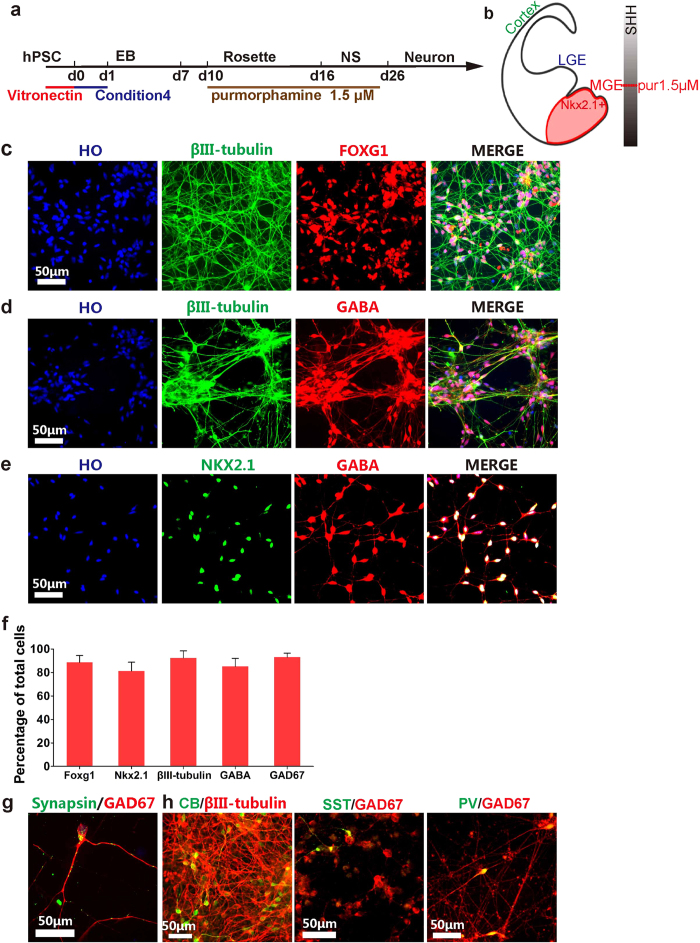
Generation of high purity GABA interneurons from hPSC under xeno-free conditions. (**a**) Timeline of direct differentiation of hPSCs to GABA interneurons under xeno-free conditions by using optimized condition 4 with high dosage of purmorphamine. (**b**) A schematic showing coronal section of human MGE (red) containing cells expressing NKX2.1 +. (**c**) At d25, nearly all the neurons (βIII-tubulin + ) co-expressed forebrain marker FOXG1. Scale bar, 50 μm. (**d**) At d30, GABA neurons co-expressed βIII-tubulin. Scale bar, 50 μm. (**e**) At d30, the majority of GABA neurons co-expressed MGE marker NKX2.1, showing the GABA neurons with MGE identity. Scale bar, 50 μm. (**f**) Quantifications of FOXG1 +, Nkx2.1 +, βIII-tubulin +, GABA + and GAD67 + cells of total cells. (**g**) At d45, nearly all the neurons expressed GAD67 and synapsin, maturation markers for GABA interneurons. Scale bar, 50 μm. (**h**) GABA interneurons expressed subtype markers Calbindin (CB), Somatostatin (SST) and Parvalbumin (PV). Scale bar, 50 μm.

GABAergic interneurons are the major inhibitory neurons in central nervous system and dysfunction of GABAergic interneurons results in neurodegenerative or psychiatric diseases^27^,^28^. Cortical GABA interneurons originate from MGE (ventral forebrain territory) (Fig. 5b) and migrate to cortex and hippocampus during development^29^. To investigate whether these GABA interneurons can be generated from hPSCs under xeno-free condition, we treated cells with 1.5 μM of Pur from d10 to d25^11^ (Fig. 5a,b, Supplementary Fig. S4a). At d28, more than 90% of hPSC-derived neurons (βIII-tubulin + ) expressed the forebrain marker FOXG1 (Fig. 5c). At d35, over 80% of cells expressed GABA, and co-expressed with βIII-tubulin (Fig. 5d, Supplementary Fig. S4b,c). The GABA neurons were NKX2.1 positive, indicating the GABA neurons were generated with MGE identity (Fig. 5e). After d45, 90% of cells expressed mature GABA-generating enzyme GAD67, together with pre-synaptic marker synapsin, demonstrating that the GABA interneurons became mature and form synaptic connections (Fig. 5g).

GABAergic interneurons are subdivided into diverse subtypes based on their expression of distinct protein markers and electrophysiological properties. Calbindin (CB), Somatostatin (SST) and Parvalbumin (PV) are the major GABAergic interneuron subtypes^30^. To identify the subtypes of GABAergic interneurons generated in the xeno-free system, all three markers were tested in the MGE cells derived under xeno-free conditions (Fig. 5h). Subtypes were identified by CB, SST, and PV-expression at d45, d55 and d85, respectively, based on previous reports utilizing feeder cells^11^,^31^. We observed each of these markers and, thus, GABAergic interneurons with diverse subtypes were generated with high efficiently under the optimized xeno-free condition.

### Astrocytes were generated under xeno-free condition

**Figure 6:**
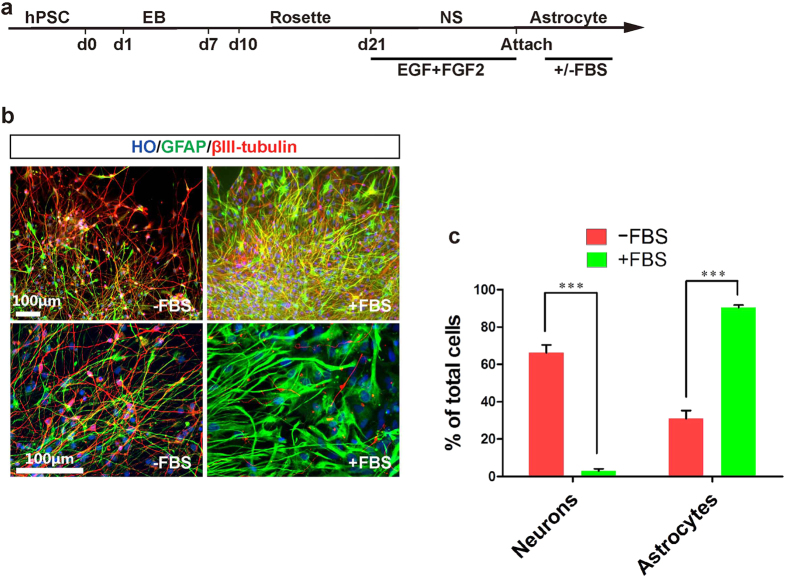
Generation of astrocytes from hPSCs under the xeno-free condition. (**a**) Timeline of direct differentiation of hPSCs to astrocytes under a xeno-free condition with the continuous treatment of bFGF and EGF for 3 months. (**b**) After differentiation for 100 days, cells expressed astrocyte marker GFAP and neuronal marker βIII-tubulin with/without the treatment of FBS. Scale bar, 100μm. (**c**) Quantification of GFAP + , βIII-tubulin + cells of total cells.

Astrocytes play an important role in the central nervous system. Many neurodegenerative and psychiatric diseases are associated with dysfunctional astrocytes. To explore whether hPSCs in xeno-free condition can differentiate to astrocytes, neural progenitors were treated with 10 ng/ml EGF and 10 ng/ml FGF2 starting at d21^32^,^33^ (Fig. 6a). With three months of continuous treatment, 90.5 ± 1.3% of cells were GFAP positive and 2.9 ± 1.2% were βIII-tubulin positive with 5% fetal bovine serum (FBS) treatment for 3 days after attachment. Without the treatment of FBS, only 31.0 ± 4.4% differentiated cells were GFAP + , but 66.3 ± 4.2% cells were βIII-tubulin + . The results suggest that FBS may enhance astrocyte fate decision (Fig. 6b,c). However, application of FBS introduces animal serum to the differentiation system. For higher astrocyte differentiation efficiency under xeno-free conditions, longer differentiation time might be required^34^.

Overall, our findings showed that hPSCs robustly generated region-specific forebrain neurons and astrocytes under xeno-free conditions, indicating that our current defined neural differentiation method is multi-potent.

## Discussion

In this study, we established an optimized paradigm for efficient differentiation of hPSCs to region-specific forebrain neurons under xeno-free conditions. Most reported neural differentiation protocols rely on animal feeder cells or undefined components, which may increase unknown risks for clinical application. In our research, the combination of E8 and vitronectin is completely chemically defined and xeno-free. More importantly, vitronectin leads hPSCs to differentiate into neurons in large scale. By applying our approach, multiple region-specific neurons and glial cells were produced with high purity without cell sorting.

In comparison with reported methods, our method has three advantages. The first advantage is the ease of using xeno-free vitronectin for the entire neural differentiation procedure, along with the exclusion of animal ingredients at each step. At the hPSC stage, the morphology of hPSCs maintained on vitronectin was similar to cells cultured on MEF. In addition, vitronectin reduces animal housing costs and avoids batch-to-batch differences when preparing MEF. In traditional neural differentiation methods, matrigel or laminin was the major substrate for neuronal attachment. However, matrigel or laminin was produced from mouse cells. In our current protocol, we applied vitronectin as the only attachment substrate through the entire procedure to exclude exogenetic factors, including hPSC maintainence, rosette attachment and neuron attachment. At neuronal attachment stage, neurons showed a stereoscopic morphology on vitronectin when compared to a “flat” morphology on matrigel or laminin. However, neurons tend to detach from vitronectin after two weeks attachment. A better xeno-free substrate or co-culture with human glial cells is required for the long-term neural attachment studies.

The second advantage of our hPSCs culture system is that multiple forebrain neuronal types are generated without adding wnt signaling inhibitors or performing cell sorting. The previously reported protocols for generating forebrain (FOXG1 + ) neurons is to use wnt inhibitor DKK1 or XAV939 in combination with dual SMAD inhibitors^7^,^12^,^31^. The addition of signaling inhibitors may cause unknown risks for the application to clinical studies. For example, DDK1 may have the potential risks to cause chronic kidney disease-associated bone and mineral disorder^35^ and inhibit neurogenesis that may contribute to cognitive decline^36^. Our previous studies had shown that FOXG1 + progenitors can be generated by default differentiation in animal feeder culture system without adding wnt signaling inhibitors or performing cell sorting^10^,^13^. Our current study modified the chemically defined neural differentiation protocol^4^,^37^ under a xeno-free condition to induce the forebrain neuronal differentiation without applying any wnt signaling inhibitors. The hPSCs generated forebrain neurons may have the potential to restore critical brain functions in central nervous system post engraftment.

The third advantage is that each step of our method is defined (Supplementary Table S2). Initially, hPSCs are cultured in xeno-free E8 medium and vitronectin. For the neural induction and specification, differentiated cells were grown in NIM, which was comprised of DMEM/F12, N2, and NEAA. N2 consists of two proteins and three compounds, which are Human Transferrin (Holo), Insulin Recombinant Full Chain, Progesterone, Putrescine and Selenite. The neurotrophic factors including EGF and FGF2 are human recombinant, and the ventral patterning factor purmorphamine is a small molecule. Therefore, all the components for neural differentiation in our current method are defined.

In summary, a robust chemical defined system for forebrain neural differentiation from hPSCs was established in our study. This xeno-free system might be used to generate near-pure specific neural cells under defined conditions. Our study provides a solid hPSC culture system and may help to move forward hPSCs related translational medical studies, including cell therapy and stem cell-based drug discovery.

## Methods

### hPSC culture and neural differentiation in a feeder free system

hPSCs (WA09, passages 20–24; 58–80; DS1, passages 30–35; DS2U, passages 35–40^18^) were maintained under feeder free conditions by coating with vitronectin (Life technology) or matrigel (BD Biosciences). For 3-4 days of maintenance in E8 medium (Life technology), hPSCs were dissociated by using EDTA (Lonza, 1 mL /well) for 1-2 minutes in 37 degrees, and reseeded at the density of 1 × 10^5^ cells per well of a 6-well plate. For neural differentiation, hPSCs were detached by dispase (Life technology) or EDTA (Lonza) to form embryoid bodies (EBs), and then cultured in neural induction medium (NIM) as previously described^11^: 500 ml of NIM contains 5 ml of N2 supplement, 5 ml of NEAA, and 490 ml of DMEM/F12. After floating for 7 days, EBs were attached on vitronectin coated surfaces. Rosette structures can be observed at d10–16. At d16, rosette colonies were detached by a 1-ml pipette manually. Non-neuroepithelial colonies can be removed at this stage. Neurospheres were continuous floated in NIM, and then dissociated by TrypLE (Life technology) and plated on vitronectin (Life technology) and poly-l-ornithine (Sigma) pre-coated coverslips for further neuronal differentiation.

### Region-specific neuronal differentiation

To differentiate cortex neurons, the process followed the default differentiation as previously described^37^. At d28, cortical progenitors were dissociated with TripLE (Life technology) and plated on vitronectin (Life technology) and poly-l-ornithine (Sigma) pre-coated coverslips at a density of 20,000 cells per coverslip. To differentiate Striatal Medium spiny neurons (MSN), 0.65 μM Purmorphine (Stemgent) was applied in NIM from d10 to d25 as previous reported^10^. To differentiate GABA interneurons, the cells were treated with 1.5 μM Purmorphine from d10 to d25 by following our previous protocol^11^. To generate astrocytes, neural progenitors were treated with EGF (10 ng/ml) and FGF2 (10 ng/ml) from d21^32^. The progenitors were dissociated into small spheres once a week. At d90, astrocytic progenitors were dissociated into single cells and plated for further identification.

### Immunocytochemistry and cellular quantification

Neurons cultured on coverslips were fixed with 4% paraformaldehyde for 30 min. After washed by phosphate buffered saline 3 times, cells were treated with 0.2% Triton for 10 minutes and blocked in 10% donkey serum for 1 hour. Cells were incubated at 4 degrees overnight in primary antibody diluted with 0.1% Triton and 5% donkey serum. On the second day, cells were incubated in secondary antibody diluted in 5% donkey serum for 1 hour at room temperature. Coverslips were mounted for fluorescent imaging. Images were acquired using an Eclipse 80i Fluorescence Microscope. Primary antibodies are list in Supplementary Table S1.

### Quantification of fluorescent images and statistics

The quantification of fluorescent images was analyzed by using Image J. At least 12 random fields were selected and more than 5,000 cells of each cell line were counted, with more than 3 independent biological replicates performed. The number of nuclei labeled by Hoechst on each field was referred to as total cell numbers. One-way ANOVA analysis of variance was performed on EB formation in different conditions. The difference between + FBS group and –FBS group were tested by paired t-test. P < 0.05 was considered to be significant. All graphical data were presented as mean ± SEM.

## Additional Information

**How to cite this article**: Yuan, F. *et al.* Efficient generation of region-specific forebrain neurons from human pluripotent stem cells under highly defined condition. *Sci. Rep.* **5**, 18550; doi: 10.1038/srep18550 (2015).

## Supplementary Material

Supplementary Information

## Figures and Tables

**Table 1:** Comparison of different conditions for embryoid body (EB) formation.

hPSC culture system	Substrate (source)	Enzyme	Day1 medium
MEF (Feeder)	MEF (Mouse)	Dispase	50% NIM & 50% E8
Condition 1 (Feeder free)	Matrigel (Mouse)	Dispase	NIM
Condition 2 (Feeder free)	Matrigel (Mouse)	Dispase	50% NIM & 50% E8
Condition 3 (Feeder free)	Vitronectin (Human)	Dispase	NIM
Condition 4 (Feeder free)	Vitronectin (Human)	Dispase	50% NIM & 50% E8
Condition 5 (Feeder free)	Vitronectin (Human)	EDTA	50% NIM & 50% E8

Six different culture systems of hPSCs with different substrates, enzymes and treatments on d1 were used for EB formation.
