# Supplementary material for: Efficient generation of region-specific forebrain neurons from human pluripotent stem cells under highly defined condition
Source: Sci Rep. 2015 Dec 16;5:18550. doi: 10.1038/srep18550 (PMC4680876; doi:10.1038/srep18550)
Supplement: Supplementary Information [file srep18550-s1.pdf]

Supplementary information

Efficient generation of region-specific forebrain neurons from human pluripotent stem cells under highly defined condition

Fang Yuan[1#], Kai-Heng Fang[1#], Shi-Ying Cao[1], Zhuang-Yin Qu[1], Qi Li[1], Robert Krencik[2], Min Xu[1], Anita Bhattacharyya[3], Yu-Wen Su[1], Dong-Ya Zhu[1*], and Yan Liu[1*]

1 Institute for Stem Cell and Neural Regeneration, School of Pharmacy, Nanjing Medical University, Nanjing, China

2 Department of Ophthalmology, University of California, San Francisco, San Francisco, CA 94143, USA

3 Waisman Center, University of Wisconsin, Madison, WI, USA

#Equal contribution

*Correspondence: yanliu@njmu.edu.cn , dyzhu@njmu.edu.cn

Supplementary Figure S1
Supplementary Figure S2
Supplementary Figure S3
Supplementary Figure S4
Supplementary Table S1
Supplementary Table S2

Supplementary Figure S1

(a) hPSCs cultured on Matrigel or VTN-NC with/without the treatment of Y27632 after passage 1 day.

(b) The morphology of hPSCs cultured on Matrigel or VTN-NC with/without the treatment of Y27632 after passage for 3 days.

(c) Bright field and immunostaining images of NANOG and SOX2 of hPSCs cultured with/without the treatment of Y27632.

(d) AP staining of hPSCs cultured with/without the treatment of Y27632.

(e) hPSCs cultured on vitronectin and in E8 medium expressed pluripotent marker SOX2 and NANOG after 20 passages.

Supplementary Figure S2

(a) Schematic of differentiating hiPSC lines DS1 and DS2U to EBs in different conditions.

(b,c) Day 1 EB formation of DS1 (b) and DS2U (c) under different conditions.

Supplementary Figure S3

(a) Timeline of direct differentiation of hiPSC DS1 and DS2U to cortical neurons in optimized xeno-free system without purmorpahmine.

(b,c) At d32, PAX6 and βIII-tubulin double positive neurons were derived from DS1 (b) and DS2U (c).

(d,e) Quantifications of PAX6+ and βIII-tubulin+ cells of total cells in DS1 (d) and DS2U (e) cell lines.

Supplementary Figure S4

(a) Timeline of direct differentiation of GABA interneurons from DS1 and DS2U iPSC lines with

the treatment of 1.5 μM purmorpahmine.

(b,c) At d35, DS1 (b) and DS2U (c) iPSCs differentiated to GABA interneurons that co-expressing GABA and βIII-tubulin.

(d,e) Quantifications of GABA+ and βIII-tubulin+ cells of total cells in DS1 (d) and (e) cell lines.

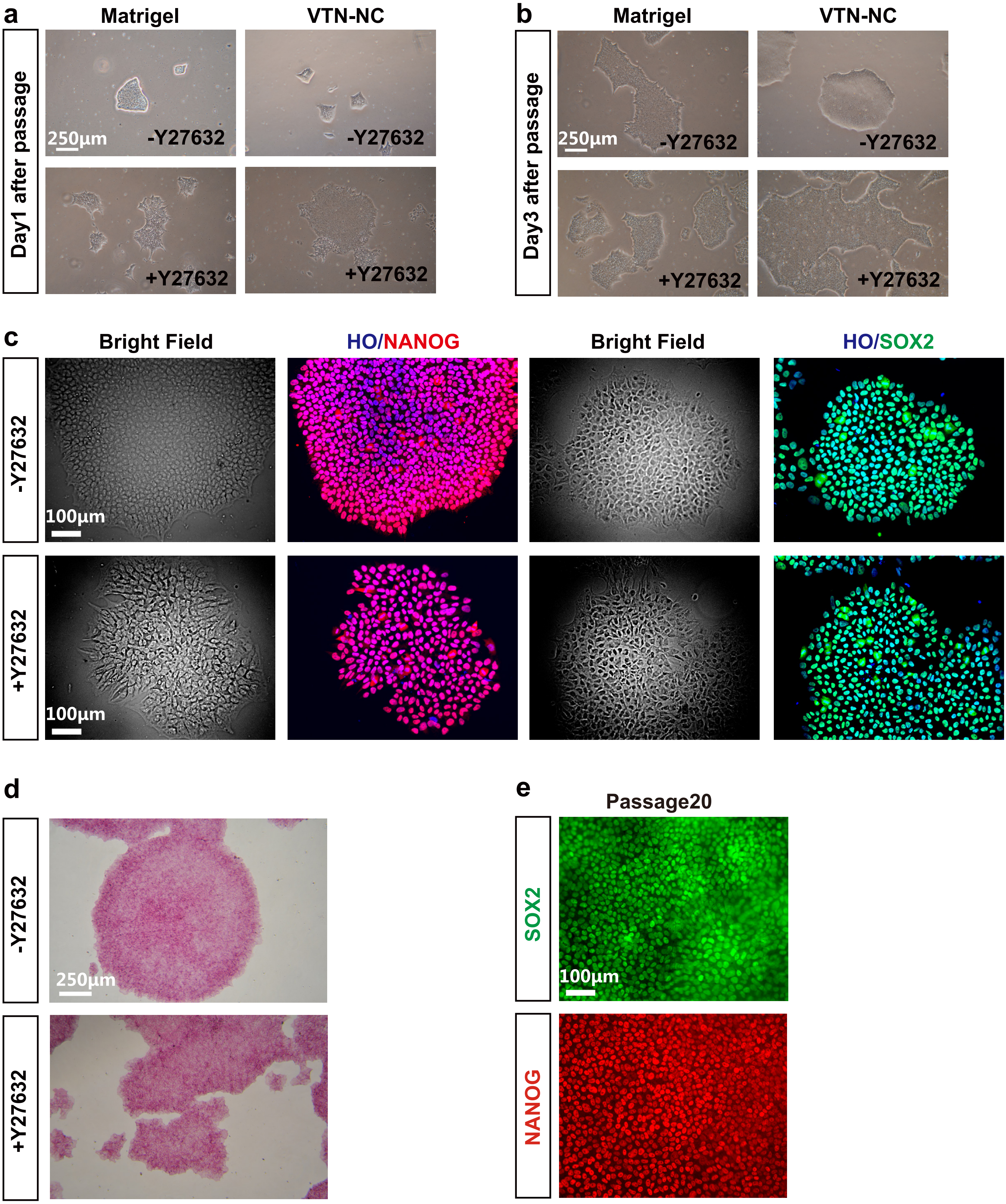

Supplementary Figure S1

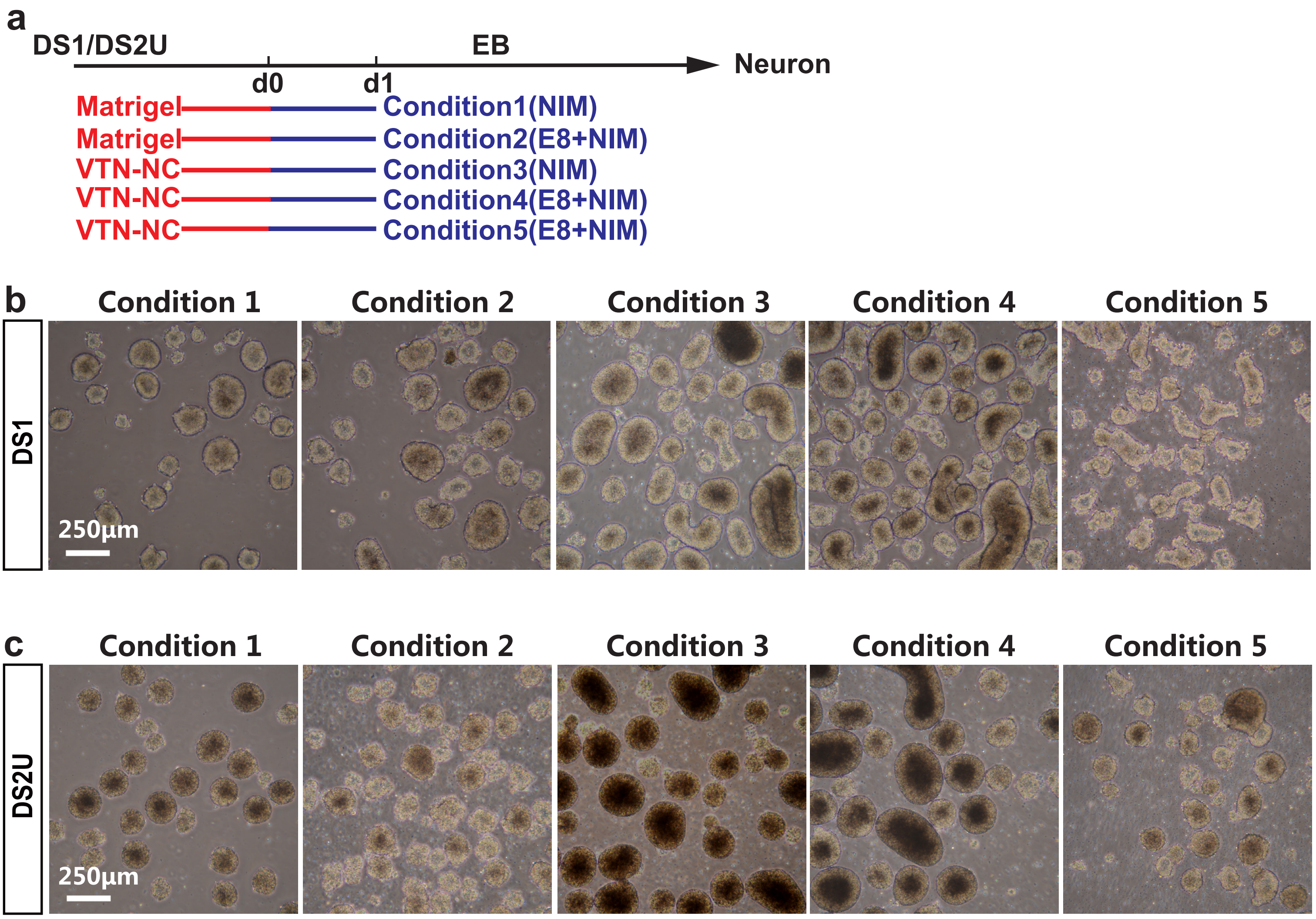

Supplementary Figure S2

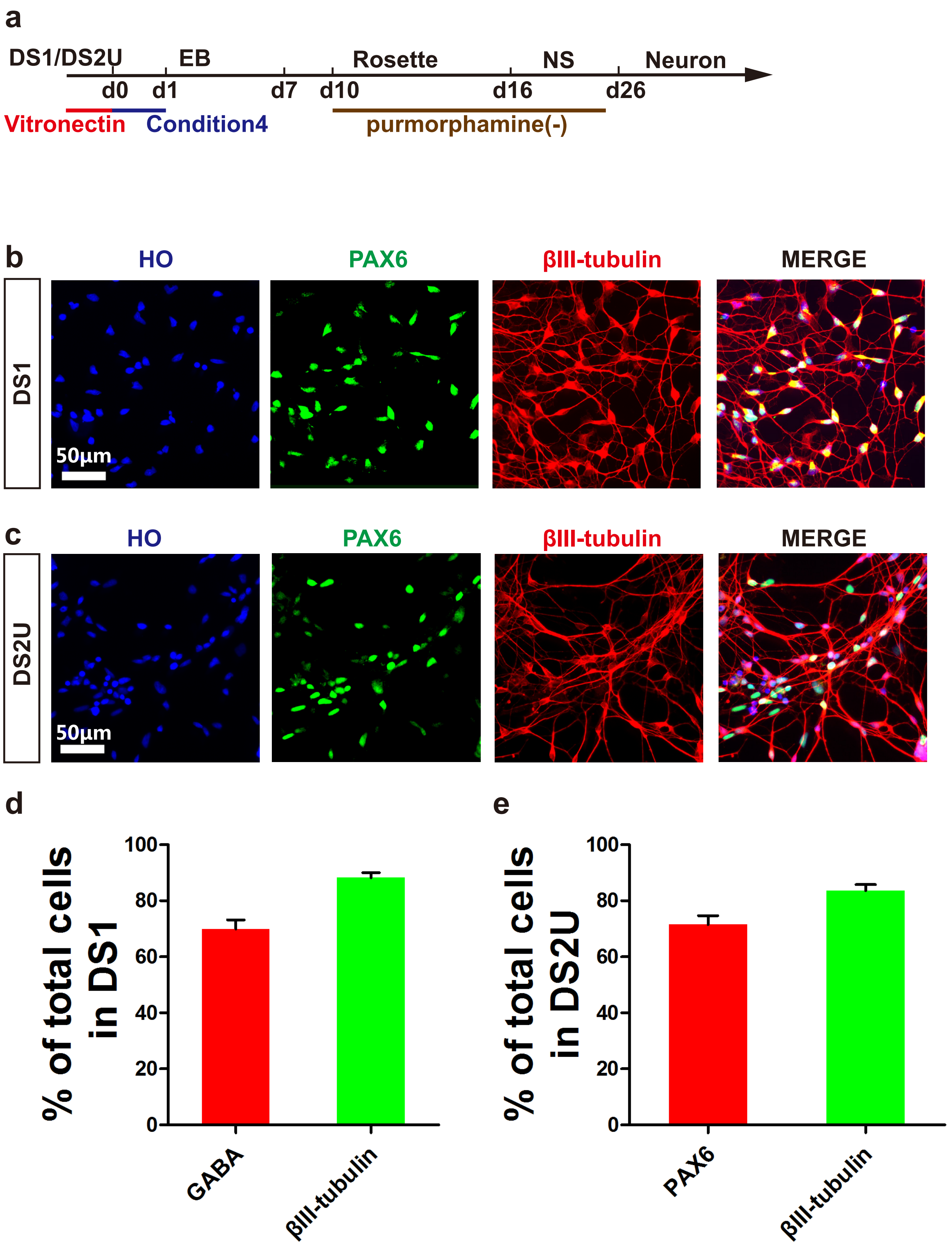

Supplementary Figure S3

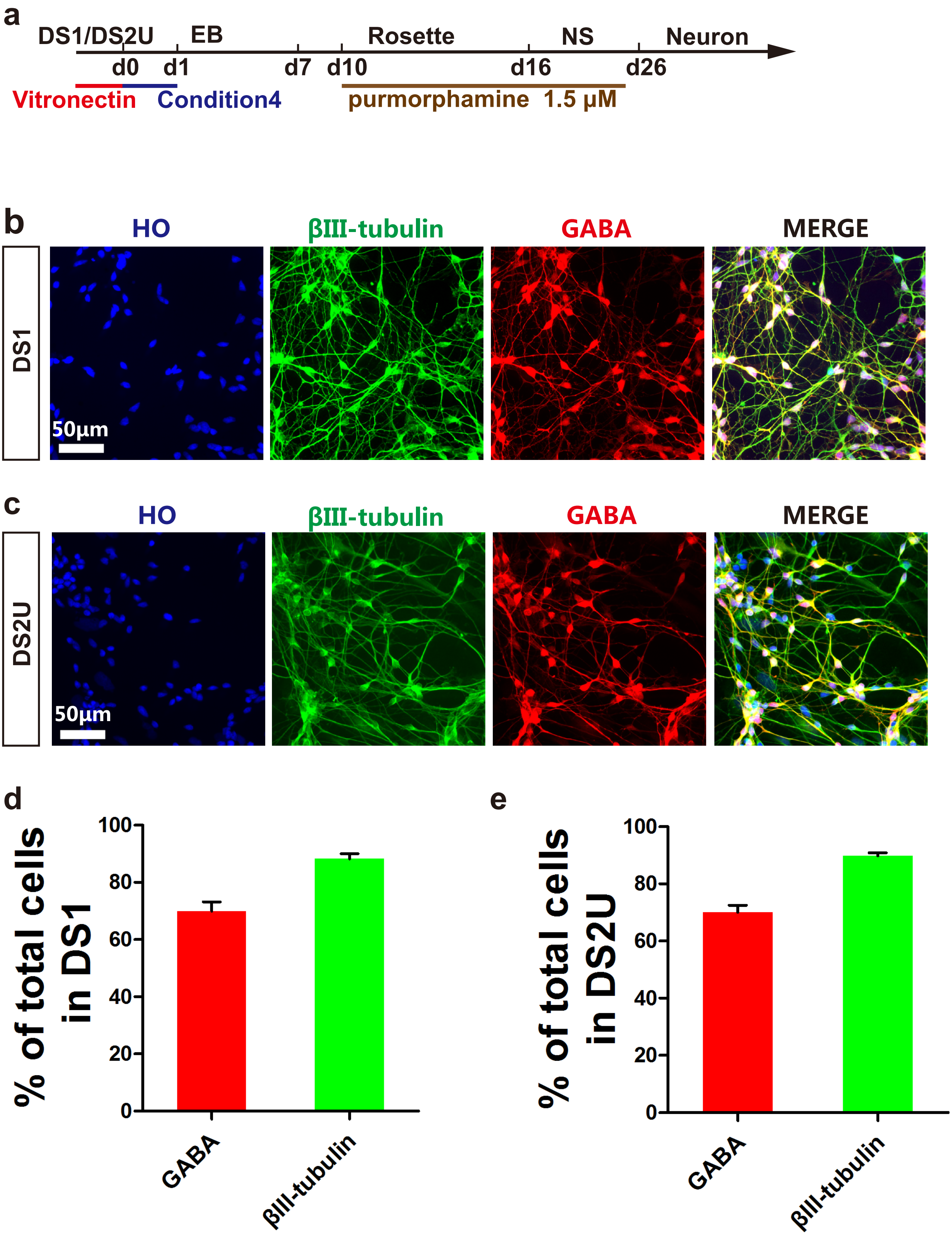

Supplementary Figure S4

Supplementary Table S1

Antibody	Isotype	Dilution	Source(cat. no.)
Calbindin(CB)	Rabbit IgG	1:1000	Abcam(AB1778)
DARPP-32	Rabbit IgG	1:2000	Epitomics(1710-1)
DCX	Rabbit IgG	1:1000	Cell Signal Technology(4604)
FOXG1	Rabbit IgG	1:1000	Abcam(AB18259)
GABA	Rabbit IgG	1:1000	Sigma-Aldrich(A2052)
GAD67	Mouse IgG	1:1000	Chemicon and Millipore(MAB5406)
GFAP	Rabbit IgG	1:1000	Dako(Z0334)
Islet1	Mouse IgG	1:500	DSHB, Iowa City, IA(40.2D6)
Map2	Mouse IgG	1:1000	Sigma-Aldrich(M1406)
Meis2	Goat IgG	1:1000	Santa Cruz biotechnology(SC-10599)
Nanog	Goat IgG	1:200	R&D Systems(AF1997)
Nkx2.1	Mouse IgG	1:500	Chemicon and Millipore(MAB5460)
Olig2	Goat IgG	1:400	Santa Cruz biotechnology(SC-19969)
PAX6	Rabbit IgG	1:1000	Covance Research Products(PRB-278P)
PAX6	Mouse IgG	1:1000	DSHB, Iowa City, IA(PAX6)
Parvalbumin(PV)	Mouse IgG	1:1000	Sigma-Aldrich(P3088)
Sox1	Goat IgG	1:1000	R&D Systems(MAB3369)
Sox2	Goat IgG	1:1000	R&D Systems(BAF2018)
Somatostatin and Receptor(SST)	Rat IgG	1:500	Chemicon and Millipore(MAB354)
Synapsin	Rabbit IgG	1:1000	MERCK(574777)
β-III Tubulin	Mouse IgG	1:2000	Sigma-Aldrich(T8660)
vGlut1	Mouse IgG	1:1000	Synaptic Systems(135311)

Supplementary Table S2

Reagent	Ingredient<br>(Working Concentration)	Feature
E8(Essential 8) Gibco,cat.no.A1517001	DMEM-F12<br>L-Ascorbic Acid<br>Selenium<br>Holo-transferrin<br>Sodium butyrate<br>Insulin<br>bFGF<br>TGFß1	xeno-free,serum free
NIM(Nerual induced medium)	DMEM/F-12(1X)<br>NEAA(100X)<br>N2 SUPPLEMENT(100X)	xeno-free,serum free
DMEM/F-12<br>Gibco, cat. no. 11330		xeno-free,serum free
NEAA(MEM non-essential amino acids solution)<br>Gibco, cat. no. 11140		xeno-free,serum free
N2 SUPPLEMENT<br>Gibco, cat. no. 17502-048	Human Transferrin (Holo)<br>Insulin Recombinant Full Chain<br>Progesterone<br>Putrescine<br>Selenite	Protein(human source)<br>Protein(human source)<br>Compound<br>Compound<br>Compound
VTN-NC(Vitronectin)<br>Gibco, cat. no. A14700	5μg/ml	Human recombination
hEGF(Epidermal Growth Factor)<br>Gibco, cat. no.PHG0311	10ng/ml	Human recombination
FGF2(Fibroblast growth factor 2)<br>R&D, cat. no. 233-FB	10ng/ml	Human recombination
